# Supplementary material for: Seeing Ɔ, remembering C: Illusions in short-term memory
Source: PLoS One. 2023 Apr 5;18(4):e0283257. doi: 10.1371/journal.pone.0283257 (PMC10075405; doi:10.1371/journal.pone.0283257)
Supplement: S1 Appendix — (DOCX) [file pone.0283257.s001.docx]

**Experiment 1**

| Model Comparison | | | | | | | | | | | |
| --- | --- | --- | --- | --- | --- | --- | --- | --- | --- | --- | --- |
| Models | | **P(M)** | | **P(M\|data)** | | **BF_M_** | | **BF_10_** | | **error %** | |
| target type + memory delay + error type + target type*error type + memory delay*error type |  | 0.053 |  | 0.500 |  | 18.002 |  | 1.000 |  |  |  |
| target type + memory delay + error type + target type*error type |  | 0.053 |  | 0.310 |  | 8.098 |  | 0.621 |  | 12.54 |  |
| target type + memory delay + error type + target type*memory delay + target type*error type + memory delay*error type + target type*memory delay*error type |  | 0.053 |  | 0.120 |  | 2.445 |  | 0.239 |  | 12.02 |  |
| target type + memory delay + error type + target type*memory delay + target type*error type + memory delay*error type |  | 0.053 |  | 0.040 |  | 0.756 |  | 0.081 |  | 11.79 |  |
| target type + memory delay + error type + target type*memory delay + target type*error type |  | 0.053 |  | 0.029 |  | 0.537 |  | 0.058 |  | 11.78 |  |
| target type + error type + target type*error type |  | 0.053 |  | 8.189e-4 |  | 0.015 |  | 0.002 |  | 11.45 |  |
| target type + memory delay + error type |  | 0.053 |  | 1.010e-17 |  | 1.818e-16 |  | 2.020e-17 |  | 11.60 |  |
| target type + memory delay + error type + memory delay*error type |  | 0.053 |  | 5.990e-18 |  | 1.078e-16 |  | 1.198e-17 |  | 11.52 |  |
| target type + memory delay + error type + target type*memory delay + memory delay*error type |  | 0.053 |  | 1.988e-18 |  | 3.578e-17 |  | 3.975e-18 |  | 70.01 |  |
| target type + memory delay + error type + target type*memory delay |  | 0.053 |  | 8.829e-19 |  | 1.589e-17 |  | 1.766e-18 |  | 11.58 |  |
| target type + error type |  | 0.053 |  | 3.097e-19 |  | 5.575e-18 |  | 6.194e-19 |  | 11.49 |  |
| memory delay + error type |  | 0.053 |  | 4.737e-30 |  | 8.527e-29 |  | 9.474e-30 |  | 11.44 |  |
| memory delay + error type + memory delay*error type |  | 0.053 |  | 1.852e-30 |  | 3.333e-29 |  | 3.703e-30 |  | 11.52 |  |
| error type |  | 0.053 |  | 5.758e-31 |  | 1.036e-29 |  | 1.151e-30 |  | 11.41 |  |
| target type + memory delay |  | 0.053 |  | 3.385e-31 |  | 6.093e-30 |  | 6.770e-31 |  | 11.48 |  |
| target type |  | 0.053 |  | 4.547e-32 |  | 8.185e-31 |  | 9.094e-32 |  | 11.41 |  |
| target type + memory delay + target type*memory delay |  | 0.053 |  | 2.953e-32 |  | 5.315e-31 |  | 5.905e-32 |  | 11.57 |  |
| memory delay |  | 0.053 |  | 7.726e-41 |  | 1.391e-39 |  | 1.545e-40 |  | 11.42 |  |
| Null model (incl. subject) |  | 0.053 |  | 2.442e-41 |  | 4.396e-40 |  | 4.885e-41 |  | 11.38 |  |
|  | | | | | | | | | | | |
| *Note.*  All models include subject | | | | | | | | | | | |

**Experiment 2**

Main ANOVA

| **Model Comparison** | | | | | | | | | | | |
| --- | --- | --- | --- | --- | --- | --- | --- | --- | --- | --- | --- |
| **Models** | | **P(M)** | | **P(M\|data)** | | **BF_M_** | | **BF_10_** | | **error %** | |
| Target Type + Memory Delay + Error type + Target Type*Memory Delay + Target Type*Error type + Memory Delay*Error type + Target Type*Memory Delay*Error type |  | 0.053 |  | 0.920 |  | 206.368 |  | 1.000 |  |  |  |
| Target Type + Memory Delay + Error type + Target Type*Memory Delay + Target Type*Error type + Memory Delay*Error type |  | 0.053 |  | 0.046 |  | 0.869 |  | 0.050 |  | 13.94 |  |
| Target Type + Memory Delay + Error type + Target Type*Memory Delay + Target Type*Error type |  | 0.053 |  | 0.026 |  | 0.479 |  | 0.028 |  | 18.84 |  |
| Target Type + Memory Delay + Error type + Target Type*Error type + Memory Delay*Error type |  | 0.053 |  | 0.006 |  | 0.103 |  | 0.006 |  | 14.70 |  |
| Target Type + Memory Delay + Error type + Target Type*Error type |  | 0.053 |  | 0.003 |  | 0.046 |  | 0.003 |  | 13.96 |  |
| Target Type + Error type + Target Type*Error type |  | 0.053 |  | 6.643e-12 |  | 1.196e-10 |  | 7.223e-12 |  | 13.96 |  |
| Target Type + Memory Delay + Error type + Target Type*Memory Delay + Memory Delay*Error type |  | 0.053 |  | 1.505e-71 |  | 2.710e-70 |  | 1.637e-71 |  | 55.50 |  |
| Target Type + Memory Delay + Error type + Target Type*Memory Delay |  | 0.053 |  | 6.615e-72 |  | 1.191e-70 |  | 7.192e-72 |  | 13.94 |  |
| Target Type + Memory Delay + Error type + Memory Delay*Error type |  | 0.053 |  | 2.151e-72 |  | 3.872e-71 |  | 2.339e-72 |  | 13.79 |  |
| Target Type + Memory Delay + Error type |  | 0.053 |  | 2.063e-72 |  | 3.714e-71 |  | 2.243e-72 |  | 13.75 |  |
| Target Type + Error type |  | 0.053 |  | 5.745e-79 |  | 1.034e-77 |  | 6.246e-79 |  | 14.17 |  |
| Target Type + Memory Delay + Target Type*Memory Delay |  | 0.053 |  | 4.149e-101 |  | 7.469e-100 |  | 4.511e-101 |  | 13.81 |  |
| Target Type + Memory Delay |  | 0.053 |  | 1.820e-101 |  | 3.276e-100 |  | 1.979e-101 |  | 13.69 |  |
| Target Type |  | 0.053 |  | 2.652e-107 |  | 4.773e-106 |  | 2.883e-107 |  | 13.66 |  |
| Memory Delay + Error type |  | 0.053 |  | 6.514e-130 |  | 1.173e-128 |  | 7.082e-130 |  | 21.02 |  |
| Memory Delay + Error type + Memory Delay*Error type |  | 0.053 |  | 3.701e-130 |  | 6.662e-129 |  | 4.024e-130 |  | 13.98 |  |
| Error type |  | 0.053 |  | 3.317e-135 |  | 5.971e-134 |  | 3.607e-135 |  | 13.65 |  |
| Memory Delay |  | 0.053 |  | 6.807e-154 |  | 1.225e-152 |  | 7.401e-154 |  | 15.40 |  |
| Null model (incl. subject) |  | 0.053 |  | 1.235e-158 |  | 2.224e-157 |  | 1.343e-158 |  | 13.62 |  |
|  | | | | | | | | | | | |
| *Note.*  All models include subject | | | | | | | | | | | |

| **Follow up ANOVA for illusory errors** | | | | | | | | | | | |
| --- | --- | --- | --- | --- | --- | --- | --- | --- | --- | --- | --- |
| **Models** | | **P(M)** | | **P(M\|data)** | | **BF_M_** | | **BF_10_** | | **error %** | |
| Memory Delay + Target Type + Memory Delay ✻  Target Type |  | 0.200 |  | 1.000 |  | 26289.122 |  | 1.000 |  |  |  |
| Memory Delay + Target Type |  | 0.200 |  | 1.521e-4 |  | 6.086e-4 |  | 1.522e-4 |  | 18.32 |  |
| Memory Delay |  | 0.200 |  | 1.911e-13 |  | 7.646e-13 |  | 1.912e-13 |  | 18.29 |  |
| Target Type |  | 0.200 |  | 1.353e-121 |  | 5.411e-121 |  | 1.353e-121 |  | 18.52 |  |
| Null model (incl. subject) |  | 0.200 |  | 2.274e-125 |  | 9.094e-125 |  | 2.274e-125 |  | 18.27 |  |
|  | | | | | | | | | | | |
| *Note.*  All models include subject | | | | | | | | | | | |

| **Follow up ANOVA for other errors** | | | | | | | | | | | |
| --- | --- | --- | --- | --- | --- | --- | --- | --- | --- | --- | --- |
| **Models** | | **P(M)** | | **P(M\|data)** | | **BF_M_** | | **BF_10_** | | **error %** | |
| Target Type |  | 0.200 |  | 0.913 |  | 41.809 |  | 1.000 |  |  |  |
| Memory Delay + Target Type |  | 0.200 |  | 0.079 |  | 0.341 |  | 0.086 |  | 2.781 |  |
| Memory Delay + Target Type + Memory Delay ✻  Target Type |  | 0.200 |  | 0.009 |  | 0.035 |  | 0.010 |  | 2.847 |  |
| Null model (incl. subject) |  | 0.200 |  | 1.572e-5 |  | 6.290e-5 |  | 1.723e-5 |  | 1.016 |  |
| Memory Delay |  | 0.200 |  | 1.321e-6 |  | 5.284e-6 |  | 1.447e-6 |  | 1.542 |  |
|  | | | | | | | | | | | |
| *Note.*  All models include subject | | | | | | | | | | | |

**Experiment 3**

| Model Comparison | | | | | | | | | | | |
| --- | --- | --- | --- | --- | --- | --- | --- | --- | --- | --- | --- |
| Models | | **P(M)** | | **P(M\|data)** | | **BF_M_** | | **BF_10_** | | **error %** | |
| target + error type + target*error type |  | 0.200 |  | 1.000 |  | 2.669e+7 |  | 1.000 |  |  |  |
| target + error type |  | 0.200 |  | 1.499e-7 |  | 5.995e-7 |  | 1.499e-7 |  | 10.586 |  |
| error type |  | 0.200 |  | 3.189e-14 |  | 1.276e-13 |  | 3.189e-14 |  | 3.595 |  |
| target |  | 0.200 |  | 1.163e-29 |  | 4.652e-29 |  | 1.163e-29 |  | 2.675 |  |
| Null model (incl. subject) |  | 0.200 |  | 1.330e-33 |  | 5.319e-33 |  | 1.330e-33 |  | 2.527 |  |
|  | | | | | | | | | | | |
| *Note.*  All models include subject | | | | | | | | | | | |

**Experiment 4**

| Model Comparison | | | | | | | | | | | |
| --- | --- | --- | --- | --- | --- | --- | --- | --- | --- | --- | --- |
| Models | | **P(M)** | | **P(M\|data)** | | **BF_M_** | | **BF_10_** | | **error %** | |
| target + memory delay + error type + target*error type + memory delay*error type |  | 0.053 |  | 0.695 |  | 41.070 |  | 1.000 |  |  |  |
| target + memory delay + error type + target*error type |  | 0.053 |  | 0.139 |  | 2.899 |  | 0.199 |  | 24.528 |  |
| target + memory delay + error type + target*memory delay + target*error type + memory delay*error type |  | 0.053 |  | 0.124 |  | 2.545 |  | 0.178 |  | 6.250 |  |
| target + memory delay + error type + target*memory delay + target*error type + memory delay*error type + target*memory delay*error type |  | 0.053 |  | 0.024 |  | 0.445 |  | 0.035 |  | 5.869 |  |
| target + memory delay + error type + target*memory delay + target*error type |  | 0.053 |  | 0.018 |  | 0.331 |  | 0.026 |  | 5.038 |  |
| target + error type + target*error type |  | 0.053 |  | 1.91e-6 |  | 3.44e-5 |  | 2.75e-6 |  | 4.158 |  |
| target + memory delay + error type + memory delay*error type |  | 0.053 |  | 1.99e-51 |  | 3.58e-50 |  | 2.86e-51 |  | 12.967 |  |
| target + memory delay + error type |  | 0.053 |  | 9.952e-52 |  | 1.791e-50 |  | 1.431e-51 |  | 6.091 |  |
| target + memory delay + error type + target*memory delay + memory delay*error type |  | 0.053 |  | 2.917e-52 |  | 5.251e-51 |  | 4.195e-52 |  | 6.277 |  |
| target + memory delay + error type + target*memory delay |  | 0.053 |  | 1.374e-52 |  | 2.473e-51 |  | 1.976e-52 |  | 3.781 |  |
| target + error type |  | 0.053 |  | 1.003e-54 |  | 1.806e-53 |  | 1.443e-54 |  | 3.807 |  |
| memory delay + error type + memory delay*error type |  | 0.053 |  | 1.339e-87 |  | 2.410e-86 |  | 1.926e-87 |  | 4.970 |  |
| memory delay + error type |  | 0.053 |  | 1.249e-87 |  | 2.248e-86 |  | 1.796e-87 |  | 3.503 |  |
| error type |  | 0.053 |  | 1.133e-89 |  | 2.040e-88 |  | 1.630e-89 |  | 3.762 |  |
| target + memory delay |  | 0.053 |  | 1.122e-91 |  | 2.019e-90 |  | 1.613e-91 |  | 3.619 |  |
| target + memory delay + target*memory delay |  | 0.053 |  | 1.866e-92 |  | 3.359e-91 |  | 2.684e-92 |  | 8.521 |  |
| target |  | 0.053 |  | 1.257e-93 |  | 2.263e-92 |  | 1.808e-93 |  | 3.345 |  |
| memory delay |  | 0.053 |  | 3.375e-119 |  | 6.074e-118 |  | 4.854e-119 |  | 3.295 |  |
| Null model (incl. subject) |  | 0.053 |  | 1.267e-120 |  | 2.280e-119 |  | 1.822e-120 |  | 3.087 |  |
|  | | | | | | | | | | | |
| *Note.*  All models include subject | | | | | | | | | | | |
